# The Role of the Microbiome in Autism: All That We Know about All That We Don’t Know

**DOI:** 10.1128/mSystems.00234-21

**Published:** 2021-04-06

**Authors:** Maude M. David

**Affiliations:** a Department of Microbiology, Oregon State University, Corvallis, Oregon, USA; b Department of Pharmaceutical Sciences, Oregon State University, Corvallis, Oregon, USA

**Keywords:** autism spectrum disorder, microbiota

## Abstract

Autism spectrum disorder (ASD) is a remarkably complex disorder influenced by both genetic and environmental factors. Numerous microbial diversity surveys conducted over the past decade have attempted to link specific ASD biomarkers to gastrointestinal tract disturbances, but results generated across cohorts and studies remain inconsistent. This commentary discusses multidirectional interactions between the host, the microbiome, and external factors germane to autism. Recent studies posit the heritability of the gut microbiome itself, confounding attempts to discern heritable from nonheritable effectors in neurodevelopmental disorders. Elucidating the ever-evolving gut microbiome’s role in modulating the ASD phenotype will most certainly require new experimental methodologies and designs. In a recent paper published in *mSystems* (J. Fouquier, N. Moreno Huizar, J. Donnelly, C. Glickman, et al., mSystems e00848-20, 2021, https://doi.org/10.1128/mSystems.00848-20), the authors describe a web of interactions by collecting samples longitudinally, analyzing cross-sectional cohorts, and recording nonbinary phenotypic measurements.

## COMMENTARY

Elucidating the countless factors contributing to the etiology of autism is incredibly challenging. Upon entering this field “late” in my career, I was then, and remain today, awestruck at both how much and how little is known about this disorder. Over the past decade, even the very definitions of the core symptoms of autism spectrum disorder (ASD) have changed. Between 2010 and 2015, both the number of known cistrons associated with autism (1) and the number of publications addressing its biomolecular foundations doubled. Autism is a staggeringly complex disorder, to state the obvious, influenced significantly by both genetic and environmental factors. While the relative proportion of impact attributed to either one of these sources continues to fluctuate, it has become clear that the ASD phenotype manifests as a consequence of both heritable and exogeneous effectors.

The enigma that is the ASD phenotype becomes even more veiled when considering the remarkably expansive and highly variable spectrum of comorbidities, both biophysical and psychiatric in nature. Gastrointestinal tract-related disturbances (2), in particular, led early researchers to posit the existence of correlations between the severity of the ASD phenotype and dysbioses of the gut microbiome. Since 2010, no fewer than nine (3) initiatives have used high-throughput sequencing to test this hypothesis, and all but one identified microbial taxa that are differentially abundant in autistic cohorts relative to controls.

Unfortunately, results generated across cohorts and studies remain inconsistent and oftentimes irreproducible. Seemingly countless confounding variables may be contributing to these disparities. For example, children affected with ASD are known to adopt more selective diets, which might alter gastrointestinal conditions and give rise to the variations observed in microbial community profiles. Ultimately, there are myriad external, highly variable factors that affect the relative health and homeostasis of the gut microbiome. Adding to the complexity, certain environmental factors associated with autism are even capable of inciting significant shifts in gut microbiome structure, thereby orchestrating complex bidirectional interactions.

Recent studies have also suggested that certain facets of the gut microbiome are heritable (4) and/or associated with specific genomic variants (5). Some researchers have gone as far as to suggest that certain autism symptoms might be heritable using microbial transplant in animal models (6). Taken collectively, these discoveries blur the thresholds that typically discern heritable from nonheritable effectors in neurodevelopmental disorders. As novel discussions are emerging on the heritability of the gut microbiome itself, new experimental methodologies and designs are required to parse out the role(s) of the constantly changing microbial consortium in modulating the ASD phenotype.

In a recent paper published in *mSystems*, Fouquier et al. (7) attempt to mitigate some of the aforementioned intrinsically complex interactions by collecting samples longitudinally, analyzing cross-sectional cohorts, and recording nonbinary phenotypic measurements. The authors exploit carefully reported social behavioral symptoms to address whether, and to what extent, the wide spectrum of ASD symptoms is associated with the microbial structure. Their results suggest that gut microbial composition metrics can be correlated with the ASD phenotype upon controlling geographic location. By leveraging curated social behavior scores over time, the authors observed that changes in lethargy/social withdrawal correlated with various degrees of alteration to the gut microbiome’s composition and diversity.

As we continue to unveil biomolecular mechanisms along the gut-brain axis, efforts to better understand the etiology of autism raise further questions. It might prove important, for example, to determine to what extent commensal microbial populations probe and respond to disturbances in the host environment. Research directed at elucidating the mechanisms by which gut microbes modulate host responses to environmental insults, both pre- and postnatal, might prove pivotal in the field of neurodevelopment.

Finally, the scientific community desperately needs to bolster the current understanding of the complex interplay between genetic-driven and heavily microbiome-influenced systems. Within this vein, we must explore whether exposure to detrimental environmental factors early in life coupled with innate genetic susceptibility might impair brain development. Addressing such questions will require novel experimental design frameworks, emerging analytical tools capable of leveraging data sets (both preexisting publicly available data sets and *de novo* data) whose unprecedented size would otherwise preclude meaningful study, and innovative techniques enabling the segregation of features of the host from those of its microbiome.
